# Mechanisms of spectral orientation in a diurnal dung beetle

**DOI:** 10.1098/rstb.2021.0287

**Published:** 2022-10-24

**Authors:** Ayse Yilmaz, Basil el Jundi, Gregor Belušič, Marcus Byrne, Emily Baird, Marie Dacke

**Affiliations:** ^1^ Department of Biology, Lund Vision Group, Lund University, 223 62 Lund, Sweden; ^2^ Department of Biology, Animal Physiology, Norwegian University of Science and Technology, 7491 Trondheim, Norway; ^3^ Department of Biology, Biotechnical Faculty, University of Ljubljana, Večna pot 111, 1000 Ljubljana, Slovenia; ^4^ School of Animal, Plant and Environmental Sciences, University of the Witwatersrand, Wits 2050, South Africa; ^5^ Department of Zoology, Division of Functional Morphology, Stockholm University, 106 91 Stockholm, Sweden

**Keywords:** Coleoptera, intensity-based orientation, photoreceptors, colour vision, spectral contrast

## Abstract

Ball rolling dung beetles use a wide range of cues to steer themselves along a fixed bearing, including the spectral gradient of scattered skylight that spans the sky. Here, we define the spectral sensitivity of the diurnal dung beetle *Kheper lamarcki* and use the information to explore the orientation performance under a range of spectral light combinations. We find that, when presented with spectrally diverse stimuli, the beetles primarily orient to the apparent brightness differences as perceived by their green photoreceptors. Under certain wavelength combinations, they also rely on spectral information to guide their movements, but the brightness and spectral directional information is never fully disentangled. Overall, our results suggest the use of a dichromatic, primitive colour vision system for the extraction of directional information from the celestial spectral gradient to support straight-line orientation.

This article is part of the theme issue ‘Understanding colour vision: molecular, physiological, neuronal and behavioural studies in arthropods’.

## 1. Introduction

Animals exploit spectral and intensity information in the sky and on the ground for several critical tasks in their everyday lives, including habitat selection, floral identification and for finding their way [1,2]. While intensity-based information can be obtained through input from only one photoreceptor type [3], the use of spectral information often requires the comparison of input from (at least) two types of photoreceptors tuned to different wavelengths [4]. If the animal's response to the spectral stimuli is independent of its intensity, it is deemed to have true colour vision [2,5]. This type of spectral analysis requires complex neuronal processing to modulate antagonistic excitatory and inhibitory neural connections between the photoreceptors and central brain regions [2,5,6]. Among insects, flower-visiting honeybees [3] and butterflies [7] are well-known examples of species with true colour vision, which allows them to perceive, learn and memorize flowers under variable illumination.

Spectral analysis can also be mediated by a few, or even just a single photoreceptor, via simple hard-wired neuronal connections. This represents a more primitive use of colour vision that is partly dependent on intensity [1,8–11]. Examples of behaviours that use this type of analysis include phototaxis toward specific wavelengths and directed movements guided by spectral gradients [2]. Ball-rolling dung beetles [12], ants [13], honeybees [14] and sandhoppers [15] are a few arthropods known to use the celestial spectral gradient to guide their movements. This gradient, which arises due to the scattering of sunlight, allows for a discrimination of the solar half of the sky—that is richer in longer wavelengths of light—from the antisolar half of the sky [13,14,16].

The diurnal ball-rolling dung beetle *Kheper lamarcki* uses a large range of celestial cues to steer straight across the savannah [17], but it is not known how they extract directional information from the spectral gradient that spans the African sky. To address this, we first characterized the spectral sensitivity of the photoreceptors of the beetle. We then set out to behaviorally define the different spectral combinations of light that could support straight-line orientation in an indoor arena. We found that *K. lamarcki* most likely rely on a primitive colour vision orientation system (rather than true colour vision) for the extraction of directional information from the celestial spectral gradient.

## 2. Material and methods

### (a) Photoreceptor recordings

The spectral sensitivities of the photoreceptors in the dorsal eyes of *Kheper lamarcki* were characterized by means of intracellular recordings in dark-adapted eyes (i.e. in animals kept in darkness for 20 min at room temperature prior to these experiments) [18]. This adaptation to the dark allows the eye to recover its sensitivity following exposure to bright light [19]. The beetles were immobilized with beeswax and resin and mounted on a goniometer that carried a micromanipulator (Sensapex, Oulu, Finland). The electrodes pulled from 1 mm diameter borosilicate capillaries on a horizontal puller (capillaries and puller P-2000; Sutter, Novato, CA, USA) were filled with 3 M KCl yielding resistance of 100–150 MΩ and inserted into the eye via a small triangular hole in the cornea. A 50 µm diameter Ag/AgCl wire inserted into the head capsule next to the eye served as a reference electrode. The signal was amplified using a SEC 10 LX amplifier (Npi electronic, Tamm, Germany), conditioned with a Cyber Amp 320 (Axon Instruments, Union City, CA, USA) and digitized via a Micro 1401 analog–digital converter (CED, Cambridge, UK). Spectral stimulation was provided with an array of LEDs (‘LED synth’; [20]), or with monochromatic light from a Xenon lamp (XBO, Cairn, UK), filtered with a monochromator (B&M, Limburg, Germany). The light sources were tuned to emit equal numbers of photons at any wavelength (‘isoquantal’ mode) and projected coaxially on the eye, with the aperture of the stimulating beam adjusted to approximately 2° with an iris diaphragm. For extracellular electroretinogram (ERG) recordings, the electrodes were pulled from 1 mm diameter borosilicate capillaries on a horizontal puller (capillaries and puller P-2000; Sutter, Novato, CA, USA). Electrodes with a approximately 1–5 µm tip were filled with insect saline (0.67% NaCl, 0.015% KCl, 0.012% CaCl_2_, 0.015% NaHCO_3_, pH 7.2) and inserted into the eye via a small triangular hole in the cornea. Spectral sensitivity was scanned with the LED synth either without additional light from the monochromator (dark-adapted), or the eye was selectively adapted by constant, coaxial illumination from the monochromator at 360, 420, 500 and 550 nm; both light sources had a wide aperture (approx. 20°). Prolonged viewing of light of a selected spectral wavelength (selective chromatic adaptation) lowers the contribution of visual cells sensitive to this wavelength to the ERG. This helps defining the spectral sensitivity maxima of the photoreceptors [21,22].

Response amplitudes from intracellular recordings and ERGs were transformed to sensitivities by means of an intensity–response function and a reverse Hill transformation [18]. For the analysis of electrophysiological data, Prism 6.0 (GraphPad, La Jolla, CA, USA) was used.

### (b) Behavioural experiments

#### (i) Animals

Experiments were performed with the diurnal dung beetle *K. lamarcki* at a field station in South Africa (24.32° E, 26.29° S) in February 2017 and 2019, and at the Department of Biology (Lund University) between February and April 2021. Beetles transported to Sweden were kept in soil-filled plastic bins in a climate-controlled room (at 26°C, and a 12 light : 12 dark regime) and fed with fresh cow dung ad libitum.

#### (ii) Experimental setup and light stimuli

To test for the capability of the beetles to orient to a single light stimulus of a given wavelength (electronic supplementary material, figure S1), two sets of LEDs 500 mA (365 nm, 395 nm, 410 nm, 450 nm, 470 nm, 505 nm, 530 nm, 590 nm; Roithner Lasertechnik, Austria) were mounted on two identical metal plates (10 × 10 cm) at an elevation of 45° (by the use of two tripods) as seen from the centre of the experimental arena. The LED plates were set 180° apart at the perimeter of a 70 cm circular wooden arena surrounded by a 30 × 30 cm high black wall. All lights were adjusted to the same photon flux of 1.0–1.23 × 10^14^ photons cm^−2^ s^−1^ measured at the centre of the arena (QE65000; Ocean Optics, Dunedin, FL, USA). Experiments were recorded using a video camera (Sony FDR-AX53 Handycam), suspended above the centre of the arena. This whole set-up was placed inside a 2.5 × 2.5 × 2.0 m blackout tent. The measured irradiance spectra of each light, in combination with the defined spectral sensitivities of the two types of photoreceptors, allowed us to calculate the absolute excitations for each photoreceptor separately. From this, the perceived relative brightness difference for UV (UV contrast), green (green contrast) and both (overall brightness) photoreceptors for each experimental condition was obtained using the Michelson contrast formula,

$$C=\;\frac{I_{\max}-I_{\min}}{I_{\max}\;+\;I_{\min}},$$

where *I*_max_ and *I*_min_ represent the maximum and minimum perceived brightness of the two lights in the experimental arena. Calculated contrast values range between 0 to 1, where 0 represents equal intensity.

#### (iii) Experimental procedure

*Orientation to a single light source.* To confirm the capability of the beetles to detect and orient to the different light stimuli used in this study, a beetle was placed alongside its dung ball in the centre of the arena—lit by one of the LED lights given above—and allowed to roll its ball to the perimeter, where the exit angle was noted. The beetle was then picked up, removed from its ball and placed back in the centre of the arena where the position of the light had simultaneously been changed by 180°. The beetle again rolled its ball to the perimeter of the arena. This procedure was repeated another four times, resulting in six exits per beetle, with the azimuth of the light changed by 180° between each exit. For statistical purposes, the exit angles of the beetles were normalized to the initial position of the light, i.e. the second, fourth and sixth exit angles of the beetles were rotated by 180°. From the angular differences between two consecutive exits of each beetle (i.e. exit angle 2 − exit angle 1, exit angle 3 − exit angle 2, etc.) the mean angular change in bearing and the length of the mean vector (*r*) were obtained. It is important to note that when initially presented with only one light cue in the arena, the dung beetles could maintain their exit angles with respect to the position of any of the light stimulus used in this study and did so with similar precision (*p* > 0.05, Friedman test). In addition, the initial bearings travelled by the beetles in relation to 365 nm, 365 nm, 450 nm and 590 nm of the light stimuli used, were not significantly different from uniformity (*p* > 0.05, Rayleigh test). For the 410 nm, 450 nm (lower intensity), 505 nm and 530 nm light stimuli, the initial bearings travelled by the beetles were still spread but tended to cluster more towards the light (*p* < 0.05, V-test) (electronic supplementary material, figure S2).

**Figure 1.**
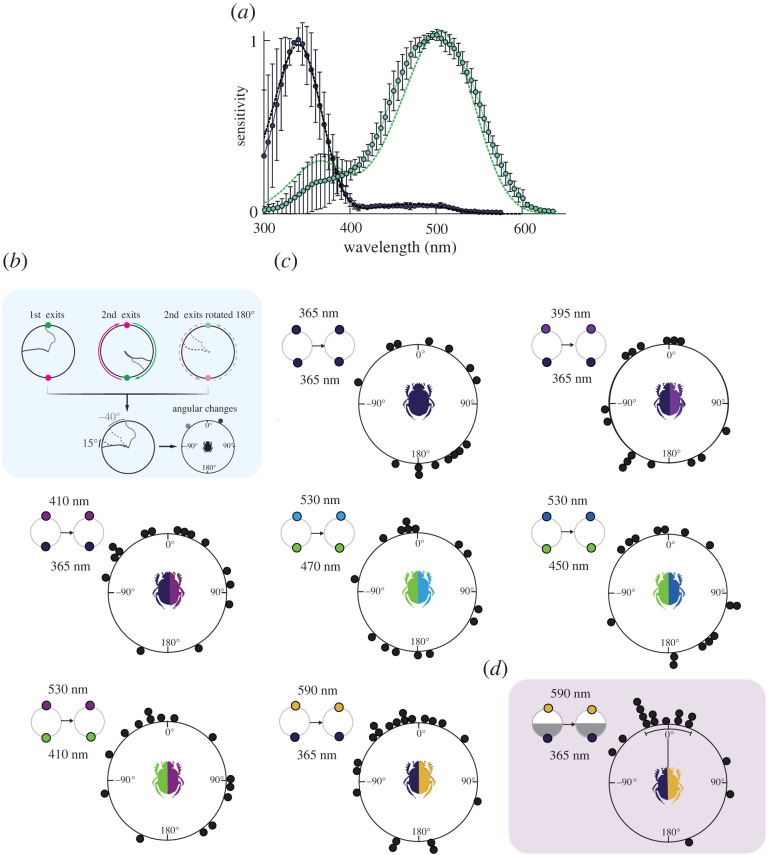
Intracellular photoreceptor recordings, and behavioural response of *Kheper lamarcki* in the presence of two spectral stimuli of similar brightness difference. (*a*) Intracellular recordings in the dorsal eye of *K. lamarcki* revealed two types of spectrally distinct photoreceptors, sensitive in the UV (*λ*_max_ ≈ 340 nm, *n* = 3, shown in black) and green (*λ*_max_ ≈ 500 nm, *n* = 6, shown in green) range of the spectrum. Dotted lines represent rhodopsin nomograms [23] with *λ*_max_ = 340 nm (black dots) and *λ*_max_ = 500 nm (green dots). Error bars indicate standard deviation. (*b*) Trajectories of two dung beetles (grey colour code) rolling a ball from the centre to the edge of the circular arena lit by two opposing lights of different wavelengths, alternating in position between the exits of the beetles. In this example, each beetle exited the arena twice, resulting in two exit angles per beetle. For statistical purposes, all exit angles of the beetles were normalized to the initial stimulus condition by rotating the exit angle by 180° with each 180° rotation of the stimulus. The angular changes between exits were then subject to further analysis. (*c*,*d*) Circular graphs represent the angular change between two consecutive exits of individual beetles in the presence of two lights, presented 180° apart. (*c*) When presented with light combinations having small differences in perceived brightness, the beetles could not maintain their original exit direction between two consecutive exits. (*d*) The angular changes between two consecutive exits were clustered around 0° when the contrast between the two sides of the arena was increased by decreasing the intensity of one of the lights. Panels with small circles represent the stimulus combinations presented to the beetles during the experiments. Grey halves in the panels indicate the experimental conditions where the intensity of one of the lights was decreased by 1 log unit. (Online version in colour.)

*Orientation to stimuli of different spectral compositions.* To evaluate the capability of the beetles to orient to different spectral contrasts (simulating a celestial contrast of different spectral compositions), each beetle was placed alongside its dung ball in the centre of the arena—lit by two lights set 180° apart—and allowed to roll its ball to the perimeter of the arena two times. In preparation for the third exit, one of the two lights was switched off. After the third exit, that light was switched on again and the beetle was allowed to exit two more times in the presence of the same stimulus combination presented during the first two exits. In preparation for the last (sixth) exit, the light that was turned off during the third exit was now presented alone. During the experiment, the relative light positions were interchanged by 180° between each exit and the order of the light presented in the third and sixth exit was randomized between individuals. To quantify each beetles' ability to orient along its initial bearing, we calculated the angular differences between two consecutive exits when (i) both light cues were available (exit angle 2 − exit angle 1, figure 1*b*) or when (ii) one of the light cues was removed during the third exit (exit angle 3 − exit angle 2 or exit angle 6 − exit angle 5). For some experiments, the intensity of one of the lights was lowered by 1 log unit (1.0–1.23 × 10^13^ photons cm^−2^ s^−1^). For statistical and illustrative purposes, the exit angles of the beetles were normalized to the initial positions of the lights (figure 1*b*).

### (c) Data analysis

The orientation response to each experimental treatment (table 1) was defined as the distribution of angular changes for different individuals between two consecutive exits from the centre of the arena. A Rayleigh test was applied to test for the uniformity of angular distributions. When different from random, a V-test was applied to determine whether the distribution of experimentally induced changes in exit angles were clustered around 0° (Oriana 3.21; Kovach Computing Services, Anglesey, UK).

**Table 1.** Statistical outcome. Mean angular changes ± circular standard deviation represents the changes in exit bearings between two consecutive rolls to different spectral stimulus combinations. We further tested if the distribution of these angular changes (see figures 1*c*, 2–4) deviated from a uniform distribution (Rayleigh test, asterisks), and if so, if they clustered around 0° (V-test).

angular change: 1st to 2nd exit			angular change: 2nd to 3rd exit			angular change: 5th to 6th exit			sample size (n)
stimuli (nm)	mean angular change (degrees) u ± s.d.	V/Rayleigh* test (p)	stimuli (nm)	mean angular change (degrees) u ± s.d.	V/Rayleigh* test (p)	stimuli (nm)	mean angular change (degrees) u ± s.d.	V/Rayleigh* test (p)	
not oriented									
365 + 365	—	*=0.072	—	—	—	—	—	—	15
365 + 395	—	*=0.44	—	—	—	—	—	—	15
365 + 410	—	*=0.06	—	—	—	—	—	—	15
365 + 590	—	*=0.07	—	—	—	—	—	—	20
530 + 410	—	*=0.60	—	—	—	—	—	—	15
530 + 450	—	*=0.71	—	—	—	—	—	—	15
530 + 470	—	*=0.94	—	—	—	—	—	—	15
oriented to intensity									
365 + 450	−3.2 ± 13.9	<0.0001	450	23.2 ± 16.2	<0.01	365	—	*=0.89	15
365 + 470	1.8 ± 10.4	<0.0001	470	23.6 ± 17.1	<0.05	365	—	*=0.62	20
365 + 505	−4.2 ± 10.6	<0.0001	505	34.6 ± 14.7	<0.01	365	—	*=0.57	25
365 + 450	−26.6 ± 12.3	<0.0001	530	56.3 ± 23.7	<0.01	395	—	*=0.71	15
365 + 505	16.3 ± 12.2	<0.0001	450^a^		*=0.38	—	—	—	15
530 + 395	2.9 ± 12.9	<0.0001	505^a^	−18.4 ± 23.0	<0.05	—	—	—	15
oriented to wavelength									
530 + 365	6.1 ± 8.2	<0.0001	530	5.23 ± 10.6	<0.0001	365	−4.9 ± 14.3	<0.0001	22

^a^intensity decreased by 1 log unit.

^b^Rayleigh test for axial data. s.d.: circular standard deviation.

## 3. Results

### (a) Photoreceptor sensitivity

Intracellular recordings in the dorsal eye of the diurnal *K. lamarcki* revealed two types of spectrally distinct photoreceptors, one maximally sensitive in the ultraviolet (UV, *λ*_max_ ≈ 340 nm, *n* = 3) and one in the green (*λ*_max_ ≈ 500 nm, *n* = 6) range of the electromagnetic spectrum (figure 1*a*). Two out of the three UV photoreceptors also displayed a tail of sensitivity (less than 5% relative to the peak at 340 nm) between 400 and 600 nm. The green photoreceptors had a slightly broader spectral sensitivity compared to the 500 nm opsin template between 300 and 600 nm, and a β-peak of sensitivity in the UV range with a variable amplitude (5–45% relative to the peak at 500 nm) (figure 1*a*).

Electroretinogram (ERG) recordings with dark and selective chromatic adaptations were also performed (for details see electronic supplementary material, figure S3). The spectral sensitivity curve obtained from the dark-adapted eyes closely matched the sensitivity curve of the green photoreceptors (electronic supplementary material, figure S3). An ERG with selective chromatic adaptation was successfully obtained in one preparation (in all other cases the adaptation was masked by the sensitivity decrease that followed a mass pigment migration). Adaptation with UV (360 nm) light selectively suppressed the sensitivity in the UV range, while adaptation with 420 nm, 500 nm and 550 nm monotonically suppressed the sensitivity of the photoreceptors between 400 and 600 nm, indicating that the retina contains only two spectral classes of photoreceptors: UV and green.

### (b) Orientation to spectral cues

#### (i) Opposing light stimuli with small differences in perceived brightness do not support orientation

When presented with two identical UV (365 nm) lights of the same physical intensity set 180° apart, the beetles were not able to maintain the same bearing between two consecutive exits (figure 1*c*). Given that the beetles in this situation were presented with an identical light environment on both sides of the arena (with a perceived brightness difference of 0) this did not come as a surprise. Next, we introduced a spectral difference of varying strengths between the two sides of the arena by presenting the 365 nm light opposite to a 395 nm, 410 nm or a 590 nm (for equal stimulation of the green channel) light, or by presenting a 530 nm light opposite to a 410 nm, 450 nm or 470 nm light. The changes in bearing between exits still did not differ from random (figure 1*c*). For these stimuli combinations, the perceived brightness difference as calculated for both photoreceptors and for the UV and green photoreceptor alone was drastically different only for the stimuli combination of 365 nm + 590 nm, that creates a green contrast as low as 0.08 (table 2). Next, we decreased the intensity of 365 nm light in this stimulus combination by 1 log unit (figure 1*d*) — resulting in a relatively higher brightness difference between the two sides of the arena (with a slight change in contrast from 0.94 to 0.95 for both PRs and an increase from 0.08 to 0.12 as calculated for only the green photoreceptor). As a result, the beetles could now orient in the same direction over several exits from the centre of the arena (*p* < 0.001, *µ* = 0.04° ± 12.4, V-test, *n* = 16, figure 1*d*). In line with previous observations of *K. lamarcki* [24,25], this demonstrates that a gradient in light intensity is an important directional cue for straight-line orientation. The inability of the beetles to initially orient under the small intensity difference as perceived by the green receptors further suggests the importance of this visual channel for intensity-based orientation. Nevertheless, it is most likely not the only determining factor since other experimental conditions, with a green contrast well above the 0.12 threshold but with a low colour contrast (for example in the 530 + 410 nm condition), did not support orientation.

**Table 2.** Colour contrast and perceived brightness differences for different stimuli combinations in the diurnal dung beetle *K. lamarcki*.

stimuli (nm)	perceived brightness difference			colour contrast
	green PRs	UV PRs	both PRs	
not oriented				
365 + 365	0	0	0	0
365 + 395	0.21	0.49	0.45	0.10
365 + 410	0.33	0.77	0.68	0.33
365 + 590	0.08	0.99	0.94	1
530 + 410	0.54	0.37	0.18	0.48
530 + 450	0.19	0.06	0.25	0.12
530 ± 470	0.11	0.47	0.36	0.14
oriented to intensity				
365 + 450	0.64	0.97	0.72	0.95
365 + 470	0.79	0.95	0.54	0.96
365 + 505	0.82	0.96	0.47	0.99
530 + 395	0.63	0.70	0.16	0.71
oriented to wavelength				
530 + 365	0.74	0.89	0.57	0.82

#### (ii) Ball-rolling beetles orient primarily with respect to the brighter stimulus

**Figure 2.**
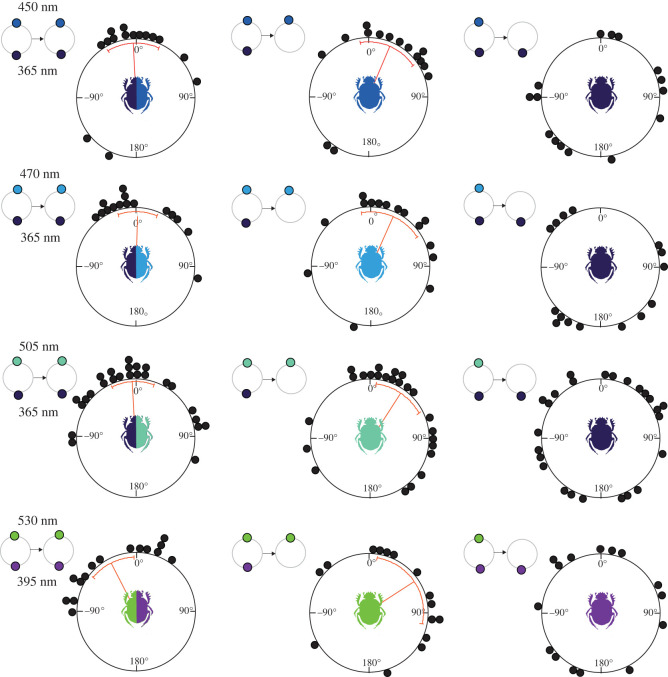
Behavioural response of *Kheper lamarcki* in the presence of two lights with spectral and brightness differences of varying intensity. Circular graphs represent the angular change between two consecutive exits of individual beetles in the presence of two lights with large differences in perceived brightness (first column) and with one of the lights presented in isolation (second and third columns)**.** The beetles could maintain their original rolling direction between two consecutive exits in the presence of both lights and in the presence of the longer wavelength light (perceived as the brighter of the two lights, second column). The beetles, however, were no longer oriented along their initial bearings when presented with only the shorter wavelength light (perceived as the dimmer light, third column). Panels with small circles represent the light combinations presented to the beetles during the experiments. Orange lines indicate mean angles, associated sectors represent the 95% confidence interval of the mean. (Online version in colour.)

Next, the 365 nm light was presented opposite to a 450 nm, 470 nm or 505 nm light, resulting in perceived brightness difference ranging from 0.47 to 0.72 for both photoreceptors and from 0.64 to 0.82 for only the green photoreceptor (table 2). The beetles’ changes of bearing between exits were now tightly clustered around 0°, indicating that they could orient using the directional information provided by these combinations of lights (figure 2). We then turned off one of the lights, placed the beetle and its ball back in the centre and let it exit again. When presented only with the ‘brighter’ long-wavelength stimulus (450 nm, 470 nm, 505 nm, 530 nm) the beetles remained oriented in the same direction as before, but when instead presented with only the ‘dimmer’ 365 nm light in isolation they could no longer consistently orient along the same bearings (figure 2 and table 1). Had the beetles used a strategy based on spectral information alone, we would expect them to be consistently oriented in the presence of either light when successively presented on its own [12,26], and if guided by the relative intensities alone, they should have changed their bearing by 180° when presented with only the dimmer cue in isolation (that would now appear as the relatively brighter cue). Our results thus suggest that the beetles—under these experimental conditions—do not rely on spectral information alone, nor relative intensity (at least not as calculated by us) as their primary cue for straight-line orientation. Instead, they appear to orient only with respect to the brighter stimulus in the arena, i.e. ignoring the relatively dimmer part of the gradient presented to them.

**Figure 3.**
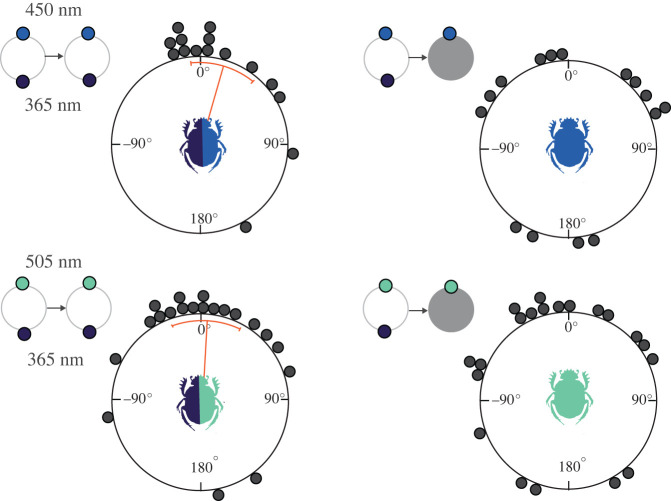
Behavioural response of *Kheper lamarcki* when the intensity of the brighter light was decreased in isolation. Circular graphs represent the angular change between two consecutive exits of individual beetles in the presence of two lights with large differences in perceived brightness (first column) and when the intensity of the brighter light was decreased by 1 log unit in isolation (second column). The beetles maintained their original exit direction when repeatedly exiting the arena in the presence of both lights, but not when presented with the dimmed version of the relatively brighter light in isolation. Panels with small circles represent the light combinations presented to the beetles during the experiments. Grey circles indicate the experimental conditions where the intensity of the light perceived as the brighter of the pair was decreased by 1 log unit. Orange lines indicate mean angles, and associated sectors represent the 95% confidence interval of the mean. (Online version in colour.)

To further evaluate this possibility, we next decreased the intensity of the 450 or 505 nm light by 1 log unit and presented them on their own, after the beetles had first exited the arena in the presence of both lights at full intensities (figure 3). The beetles were now—to our surprise—no longer able to orient along their initial bearings when presented with either of the two lights. It is important to note that the even if the beetles now exited along a different bearing, they still rolled along straight paths, demonstrating that they could reliably detect the dimmed lights. The changes in exits recorded for beetles in the presence of either of the dimmed lights suggest that the beetles also take the absolute intensity of the brighter side of the arena into account when guiding their straight-line exits from the centre of the arena.

As soon as the two brighter lights were reintroduced into the arena, the beetles again oriented along their initial bearings (*p* < 0.001, µ = 0.29 ± 17.2°, V-test, *n* = 35). This demonstrated that the beetles, that just appeared to have chosen a new, random bearing, could still orient along their initial bearings in the presence of the relevant stimuli.

#### (iii) A primitive form of spectral orientation restricted to specific wavelengths

**Figure 4.**
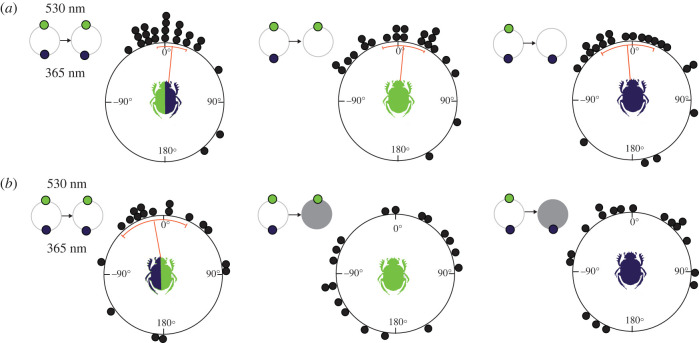
Behavioural response of *Kheper lamarcki* in the presence of 365 nm and 530 nm light stimuli. (*a*) Circular graphs represent the angular difference between two consecutive exits of individual beetles in the presence of two light stimuli with high differences in perceived brightness (first column) and when the intensity of the brighter stimulus was decreased in isolation (second column). The beetles could maintain their original exit direction between two consecutive exits, not only in the presence of two light stimuli but also when the brighter (530 nm) and dimmer (365 nm) stimuli were successively presented in isolation. (*b*) The beetles were, however, no longer oriented along their initial bearings when presented with either dimmed cue in isolation (second and third column). Panels with small circles represent the stimulus combinations presented to the beetles during the experiments. Grey circles indicate the experimental conditions where the intensity of the light stimulus was decreased by 1 log unit. Orange lines indicate the mean angles, and the associated sectors represent the 95% confidence interval of the mean. (Online version in colour.)

Interestingly, when presenting the beetles with a 365 nm light on one side and a 530 nm light on the other (with a perceived brightness difference of 0.16 for both photoreceptors and 0.63 for the green photoreceptor), we obtained a different result from that of all other stimulus combinations (figure 4). The beetles were now not only well oriented in the presence of both lights and with the relatively brighter 530 nm light when successively presented in isolation, but also when successively presented with the relatively dimmer 365 nm light in isolation (figure 4*a*). These results suggest that the beetle's orientation was now either mediated by spectral information, absolute (perceived) intensity or by a combination of the two. To solve this issue, we decreased the intensity of each stimulus (365 nm and 530 nm) by 1 log unit and presented each light on its own, again after first letting the beetles exit in the presence of both lights. As a result, the beetles were no longer able to orient along their initial bearings under either light (figure 4*b*). As a true colour vision system should be stable to these relatively small changes in intensity, this outcome suggests that the beetles do not employ true colour vision for orientation. While this single result does not allow us to rule out the use of absolute, perceived intensity as a cue for ‘spectral’ orientation, the inability of the beetles to orient to the 365 nm light only (after first being presented with this light in combination with a longer wavelength light) in other experiments (figure 2) support our conclusion to eliminate this possibility, at least as a general strategy for orientation. The combined results from this study suggest that *K. lamarcki* uses a primitive form of spectral analysis—not fully decoupled from the intensity—as the underlying mechanism for straight-line orientation.

## 4. Discussion

The diurnal dung beetle *K. lamarcki* seems to rely on a dichromatic colour vision system with two spectrally distinct photoreceptor types: one sensitive in the short-wavelength range (UV, *λ*_max_ ≈ 340 nm) and one in the long-wavelength range (green, *λ*_max_ ≈ 500 nm) of the electromagnetic spectrum (figure 1*a*). The great majority of scarabs possess the same set of photoreceptors [27,28], and an additional third photoreceptor type has so far only been observed in flower-visiting species [29]. This common lack of a functional blue photoreceptor is suggested to be an adaptation to spectrally attenuated visual habitats, low-intensity light levels or a limited use of landmarks for navigation [27,30–32]. The savannah-living ball-rolling beetle *K. lamarcki* does indeed steer without the use of landmarks [33] and many of its close relatives fly at night [34], but its sunlit habitat is in no way deprived of colour. Like dichromatic ants and sandhoppers [13,15,35,36], these ball-rolling beetles are also known to orient to the celestial spectral gradient that spans the sky [12].

### (a) Intensity-based cues for straight-line orientation are primarily analysed by the green channel

To create a simplified and precisely controlled gradient of colour and intensity in the dorsal view of the ball-rolling beetle, two lights of different spectral properties—but adjusted to identical physical intensities—were presented at an elevation of 45° from opposite sides of a featureless arena (see table 1 for the 16 combinations of wavelengths and intensities used). If one of the two lights of the four stimuli combinations that supported straight-line orientation (table 1 and figure 2) was suddenly removed, the beetles maintained their former headings exclusively in the presence of the light perceived as the brighter. By contrast, in the sole presence of the relatively dimmer light—that when presented on its own naturally defines the brightest part of the arena—the distribution of headings deviated in all directions (except for the 365 + 530 nm combination, discussed further below). This indicates that the beetles did not simply orient to the brightest part of the arena. Had they done so, we would have expected the majority of the beetles to change their bearings by about 180°. Taken together, our results rather suggest that, under these experimental conditions, where the beetles primarily oriented with respect to the light perceived as the brighter of the pair, they also took its absolute perceived intensity and/or spectral characteristics into account.

We further found that the beetles which failed to orient along their initial bearings in the presence of only the relatively dimmer light, were still able to roll their balls of dung along their initial bearings again when the brighter stimulus was reintroduced into the setup. This is likely because the beetles' paths are guided by a stored celestial snapshot [26]—acquired when they set out from the dung pat or, in this case, the first exit from the centre of the arena—in which they register and learn the spatial distribution of available directional cues. In this study, the beetles were apparently not able to match the dimmer light to this initial snapshot when subsequently presented on its own (for which the relative intensity now changed from dim to bright, while its absolute intensity and spectral characteristics remained unchanged). Note that when initially presented on their own, all light stimuli used in this study can support straight-line orientation in *K. lamarcki* (electronic supplementary material, figure S2). The observation that the beetles failed to orient along their initial bearings in the sole presence of the relatively dimmer light (of the initially paired combination) thus suggests that relative perceived intensity is also recorded in the snapshot of the beetle.

While our experiments do not reveal exactly how the beetles process the intensity component of the spectral stimuli used for orientation, the tight correlation between their orientation performance and the increased perceived brightness differences in the arena, as calculated for the green photoreceptors, suggests that their analysis of light intensity is supported mainly by the green channel. This has also been shown in honeybees, which differentiate between the relative intensities of flowers when foraging [3]. It is also interesting to note that, when presented with the 365 + 590 nm combination of stimuli with a high spectral and UV contrast but a low green contrast (table 2), the beetles were not able to consistently orient along a given bearing. This gives a strong indication that (i) the beetles orient to differences in intensity over colour contrast and (ii) it is primarily the green photoreceptors that dominate this analysis.

### (b) A spectral ‘gradient’ from UV to green provides spectral information for orientation

Out of the 11 different spectral combinations of lights presented to them (table 2), *K. lamarcki* seemed to rely on spectral information for orientation only when presented with a stimulus combination of 365 + 530 nm. The beetles were now not only successfully oriented along their initial bearings in the presence of both light stimuli, but also when either light was successively presented in isolation (figure 4*a*). Earlier studies performed on *K. lamarcki* using exactly the same stimulus combination, came to the same conclusion [12,26]. However, as soon as we presented a 1 log unit dimmed version of either light in isolation, again after first having let the beetle roll in the presence of the brighter lights, the beetles still rolled along straight and well-directed paths (demonstrating that they could detect the dimmed light) but were no longer oriented along their initial bearings (figure 4*b*). As a colour vision system should support behaviour, independent of the changes in intensity [1,2,8,37], our results suggest that dung beetles do not rely on true colour vision for celestial orientation. However, the ability of the beetles to maintain a consistent orientation in the sole presence of either light, but with unchanged intensity conditions, opens the possibility of a more primitive colour vision with the opponent chromatic interaction of the UV and green photoreceptors as a mechanism for spectral orientation. Similar antagonistic opponent mechanisms are known to support host-finding behaviours of aphids [38,39] and host-plant detection in whiteflies [11]. In summary, our results suggest that *K. lamarcki* relies on a primitive mechanism for spectral analysis coupled to intensity to extract directional information from the simple gradients of colour presented to them in this study but does not exclude the use of true colour vision in different behavioural contexts.

It remains, however, an open question why the dung beetles were only guided by spectral directional information in the 365 + 530 nm combination, and not for example in the 365 + 505 nm that more closely matched the peak values of their two photoreceptors (UV, *λ*_max_ ≈ 340 nm and green, *λ*_max_ ≈ 500 nm). Unfortunately, our current data do not allow us to answer this question, but it is possible that with the 365 + 530 nm combination, the excitation ratio fed into the colour opponent system was just right to facilitate antagonistic neural processing [11] and, as a consequence, the beetles relied more heavily on spectral directional information for orientation than in the other experimental conditions. Such flexible use of compass cue preferences relative to the prevailing visual scenery is well documented within the heading compass network of the beetles [40,41]. However, as soon as the intensity of the light presented in isolation was decreased by 1 log unit, the beetles could no longer orient to it (figure 4*b*), again demonstrating that intensity still plays a role when orienting with spectral information.

### (c) Celestial orientation in the field

When rolling balls across the savannah, a primitive mechanism for spectral analysis coupled to intensity should still allow dung beetles to extract directional information from the spectral gradient that spans the sunlit sky. There, the continuous decrease in long-wavelength light present in the sky as one turns away from the sun is naturally accompanied by a continuous decrease in overall light intensity. Similar to ants [13,42], honeybees [14] and sandhoppers [15], the dung beetles take advantage of both these gradients to find their way [12,24,25]. To avoid any possible influences of terrestrial structures on the directional analysis of these gradients, it might even be advantageous to *not* decouple these two qualities of light. While the beetles will indeed interpret a bright, green light as the sun when tested in the lab [43] it remains to be verified if they—like bees [13]—also interpret a UV light spot as the anti-sun.

On a clear day, the celestial gradients of colour and intensity are also accompanied by a pattern of polarized skylight and a clear view of the sun. For the ball-rolling beetles, these two latter cues support a high precision of orientation [26] and, when the sun or polarized light are available, the celestial gradients of light seem to play only a minor role in the compass network of the beetles [26,44]. However, under overcast weather, when the sun is hidden behind clouds (and the degree of polarization weakens [45]), the sandhopper *Talitrus saltator* can still find guidance in the celestial spectral gradient alone [15]. While we know that these beetles are unable to orientate under heavily overcast conditions [46], we will next turn our focus to its possible role under lighter cloud covers. While this is a rare situation for savannah-living species, forest-living dung beetles are faced with this type of sky on a frequent basis.

## Data Availability

The datasets supporting this article have been uploaded as part of the electronic supplementary material [47].
